# Editorial

**DOI:** 10.4102/sajhivmed.v20i1.1037

**Published:** 2019-12-02

**Authors:** David C. Spencer

**Affiliations:** 1 Division of Infectious Diseases, Department of Medicine, Helen Joseph Hospital, University of the Witwatersrand, Johannesburg, South Africa

Dear Members of the Southern African HIV Clinicians’ Society and Readers of the *Southern African Journal of HIV Medicine*.

This is an update of articles published in the SAJHIVMED between May and July 2019. We hope that by going through this summary you will visit the journal and look at the published material yourself. The articles address contemporary and regional issues in HIV medicine. The topics speak to all aspects of the epidemic: epidemiology, public health, prevention, clinical medicine, tuberculosis and opportunistic diseases, management guidelines, opinion pieces, editorials and case reports. For the teachers, trainers, healthcare managers and administrators among us, there is a wealth of local information in these papers. Please acknowledge our talented researchers by reading what they write. I am sure you will want to thank the authors, our reviewers and our publishers (AOSIS). I wish you an enjoyable read with the *Southern African Journal of HIV Medicine*.

**May 2019**

1. Aigbodion SJ, Motara F, Laher AE. Occupational blood and body fluid exposures to human immunodeficiency virus post-exposure prophylaxis. South Afr J HIV Med. 2019;20(1):a958. https://doi.org/10.4102/sajhivmed.v20i1.958

**Recommended reading**: This is a must-read for all practising in the southern African region and for final-year medical students in particular who will soon become interns and nurses who are already working on the wards of our hospitals.

This is a well-written, descriptive (anonymous questionnaire), cross-sectional study reflecting the experience over 2 years of interns exposed to HIV-positive blood or body fluids in four large Gauteng public hospitals affiliated to the University of the Witwatersrand. The study data were collected at the end of 2017 from 175 doctors aged 24–30 years who collectively provided *n* = 182 incidence reports of occupational exposure to HIV, served as the sample for the study. The prevalence of exposure was more or less identical whether the intern was gaining experience in surgery, OBGYN or medicine. More than a fifth (*n* = 30, 22%) had more than one exposure. While most initiated post-exposure therapy within 24 h (79% on first-exposures, and 89% on their third exposure) only two-thirds viz. 63% (first exposure) and 62.5% (third exposure) completed the 28-day antiretroviral (ART) course. (How many started ART within 2–4 h?) Taking all exposures into account, the full 28-day course was completed by only *n* = 51 (36.2%). A third took only two antiretrovirals (ARVs) as post-exposure prophylaxis (PEP). A total of *n* = 33 (18%), were unaware of alternative treatment options. Two (1.1%) seroconversions are reported and documented. Are the number of ARVs used in PEP important? ‘There is no evidence to suggest that a three-drug regimen is superior to a two drug regimen’. The authors do not seem to think so. They have a point. But not all ARVs are equal in potency nor do all offer the same level (barrier) to viral resistance. Three-drug regimens, usually boosted protease inhibitor (bPI) based, reflect a time when ARVs were generally less potent or more toxic than now and when regimens that combined different classes of ARV demonstrated greatest efficacy. The authors acknowledge limitations: the cross-sectional and retrospective nature of the data, the limited range of the questions and insufficient data on exposure to ART-resistant virus. PEP studies cannot be randomised controlled trials nor can they be placebo-controlled. These studies are therefore important despite their limitations.

**PS:** The 2019 Southern African HIV Clinicians’ PEP guidelines are under preparation and will be available in this journal early in 2020. If I was a prospective intern, I would be hoping that my senior in the unit would give me complimentary copies of these two articles before I started work! NB The society’s last PEP guidelines were formulated in 2015. A more up-to-date edition is recommended.

2. Mndzebele S, Matonyane LG. Sexual behaviours, awareness and perceptions towards voluntary medical male circumcision among students in Dr Kenneth Kaunda District, South Africa. South Afr J HIV Med. 2019;20(1):a846. https://doi.org/10.4102/sajhivmed.v20i1.846

**Editor’s comment:** This cross-sectional, questionnaire-based, descriptive study on attitudes of young male South African college students towards medical male circumcision revealed that the 351 participants had high levels of knowledge and understanding of the procedure and its benefits. Many of the students were themselves circumcised viz. 77.6%, and had chosen to have MMC (78.2%). Is this a changing trend in SA?

3. Edet A, Akinsola HA, Bessong PA. Virologic and immunologic responses of patients on highly active antiretroviral therapy in a rural community health centre in Limpopo, South Africa: A retrospective study. South Afr J HIV Med. 2019;20(1):a818. https://doi.org/10.4102/sajhivmed.v20i1.818

**Editor’s comment: Recommended reading – a study that ought to be read by all**. This retrospective analysis records data spanning 12 years (2004–2016) and asks the question, ‘what are the long-term immunologic (CD4) and virologic (viral load) consequences of ART in a rural region of South Africa’. ‘Is (rural) SA on the road to achieving the Joint United Nations Programme on HIV and AIDS (UNAIDS) 90-90-90 goals?’ ‘Will universal ART in this rural region offer the reward of “Treatment as Prevention,” that is long-term viral suppression and no further viral transmission?’ The study is well set-out and very easy to follow. A total of 1247 patients were followed. All had to have been on ART for a minimum of 6 months. The analysis suggests that achieving the 90-90-90 goal is unlikely: viral suppression *at* < 50 cp/ml after 6 months, 12 months, 24 months, 36 months and 132 months after starting ART is 64%, 70%, 70%, 69% and 94%, respectively. The last percentage can be ignored as only 16 patients were available to be assessed at 12 years. *n* = 882 (59%) recorded two or more consecutive viral loads < 50 cp/ml? But only 14% had persistent viral load suppression *at* < 50 cp/ml for the initial 54 months of the study. Will rural SA reach the UNAIDS 90-90-90 goal by 2020? Will viral transmission come to an end any time soon? The answers are all too obvious after reading this intelligent and readable article.

**PS.** If you teach HIV medicine, this is a useful study to have in your repertoire.

4. Essa R, Maharaj S, Hari K, Motakef S. Tonsil histopathology in HIV-infected versus HIV-uninfected adults. South Afr J HIV Med. 2019;20(1):a936. https://doi.org/10.4102/sajhivmed.v20i1.936

**Editor’s comment:** This is a retrospective histological review covering 10 years (2005–2015) of adult tonsillectomies in the Department of Otorhinolaryngology (ENT)/Head and Neck Surgery at the University of the Witwatersrand in Johannesburg. Although the results are divided into two categories, viz. HIV-infected (*n* = 84) and HIV-uninfected (*n* = 74), the study is largely descriptive and there is no attempt to link findings with patient demographics, CD4 results, viral loads and the clinical details of the cases. Statistical and comparative data are, for the most part, left unexplored. Although reactive lymphoid hyperplasia was the most frequent histological finding in both arms viz. 77% in HIV-positive and negative, no data are provided to explain the cause of the reactive hyperplasia in the HIV-uninfected group. Were other viruses, for example, EBV, CMV, HPV, HHV8 and others implicated?

5. Moorhouse M, Cohen K. The role of rilpivirine in Southern Africa. South Afr J HIV Med. 2019;20(1):a825. https://doi.org/10.4102/sajhivmed.v20i1.825

**Editor’s comment: Recommended reading**. This is a comprehensive review of the role of rilpivirine (RPV) in the context of ART, pre-exposure (PrEP) and PEP in public sector programmes of low-and middle-income countries (LMICs). The authors address regional ART-issues that will impact on RPV use viz. irregular or unavailable viral load testing, RPV’s loss of efficacy in the context of high (baseline) viral loads, rifampicin and RPV (tuberculosis therapy), RPV and dolutegravir (DTG), other drug–drug interactions and long-acting RPV in future PrEP and PEP programmes. The findings are quite interesting and relevant.

**PS:** For those who are writing exams later this year or in early 2020, this is a must-read. But for all of us, this is a nuts-and-bolts review that deserves to be read.

**June 2019**

6. Manjengwa PA, Mangold K, Musekiwa A, Kuonza LR. Cognitive and behavioural determinants of multiple sexual partnerships and condom use in South Africa: Results of a national survey. South Afr J HIV Med. 2019;20(1):a868. https://doi.org/10.4102/sajhivmed.v20i1.868

**Editor’s comment: Recommended reading**. This is another well-crafted paper. It is a cross-sectional and descriptive report that draws upon the Third National HIV Survey of 2012. The researchers ask why South Africans continue to take risks. Two high-risk groups are defined: those with multiple sexual partners (MSPs) and those who do not use condoms consistently viz. non-condom users (nCU). The parent survey included 10 034 people. This study employs a sample of 6061 people who provided information about sexual behaviour in the preceding 12 months. **Thirteen per cent** (*n* = 744/6061) **were MSPs** and **53**% (*n* = 3158/6039) **were nCUs**. Respondents in the MSP group indicated that ‘perceived benefits’ (adjusted odds ratios, aOR = 2.16) and a related issue, intergenerational sex (aOR = 2.14), and non-susceptibility to HIV, that is irrational beliefs like ‘it won’t happen to me’, lay behind their actions. Similar reasoning defined the responses of the nCUs: perceived benefits (aOR = 1.25), non-susceptibility to HIV (aOR = 1.6) and my ‘personal belief’ (aOR = 1.35). These irrational and dangerous responses jeopardise attempts to bring the epidemic to an end. I recommend this paper for further reading. Is our community aware of these data? And would that make any difference?

7. Sharp J, Wilkinson L, Cox V, Cragg C, Van Cutsem G, Grimsrud A. Outcomes of patients enrolled in an antiretroviral adherence club with recent viral suppression after experiencing elevated viral loads. South Afr J HIV Med. 2019;20(1):a905. https://doi.org/10.4102/sajhivmed.v20i1.905

**Editor’s comment:** Patients at high risk of treatment failure (*n* = 165) were enrolled in an adherence club rather than being retained in their parent treatment facility viz. Ubuntu Clinic, Khayelitsha, Western Cape, SA. Most of the patients (81.8%) were women. Enrolment started in 2012–2014 and the study ended in mid-2015. Data were analysed retrospectively. The target population had demonstrated difficulty with ART adherence prior to their integration into the study. The outcomes with regard to both retention in care viz. 98% (6 months), 95% (12 months) and 89% (18 months) and viral suppression viz. < 400 cp/mL, 90% (6 months), 84% (12 months) and 75% (18 months) are comparable with those of clinic-based adherence studies elsewhere. This is a clearly written article with an important message: with commitment from patients and the caregiver, high-risk patients can be accommodated within a ‘differentiated’ model of ART delivery. Limitations? I would watch the 18-month numbers down the line and would want data that are more inclusive of men in the Western Cape. Despite the absence of a control group, the retrospective nature of the study and the incomplete tracing of those lost to follow-up, it nevertheless is a good read.

8. Coetzee M, Delport SD. Peripartum HIV infection in very low birth weight infants fed ‘raw’ mother’s own milk. South Afr J HIV Med. 2019;20(1):a912. https://doi.org/10.4102/sajhivmed.v20i1.912

**Editor’s comment: An important paper to read.** This is another retrospective study that identified 80 very low birth weight (< 1500 g) infants born to HIV-infected mothers between 2010 and 2013. The authors are paediatricians from Kalafong Hospital in Pretoria. Two (2.5%) of the 80 infants tested HIV-positive after birth. Neither mother had been on ART during pregnancy. Sixty-three infants (79%) had been exposed to maternal ART during pregnancy. None tested positive at the 4–6 week follow-up clinic visit. The two infants who were infected belonged to a group of 17 ART-naïve mothers. All the newborns received nevirapine prophylaxis. All were given mother’s milk – ‘raw mother’s milk’. A small group (*n* = 21/80, 26%) required additional breast milk given by donors. When did the two acquire infection? Was the ‘raw’ breast milk the source of virus or infection? The authors argue not. Both children developed clinical signs of ‘acute’ HIV seroconversion shortly after birth. They tested HIV-polymerase chain reaction (PCR) positive on day 9 and day 20 respectively. Neither had been tested at birth. The authors did a good job of taking the reader through the complicated evolution of mother-to-child HIV prevention in the last decade in South Africa. Current goal posts viz. birth testing of all exposed infants and universal HIV testing and treatment of all, ought to pre-empt the loop-holes identified in this study. This paper is an important read. Very low birth weight newborns are at-risk people who require focused care.

9. Lazarus E, Otwombe K, Dietrich J, et al. Vaginal practices among women at risk for HIV acquisition in Soweto, South Africa. South Afr J HIV Med. 2019;20(1):a866. https://doi.org/10.4102/sajhivmed.v20i1.866

**Editor’s comment:** This is a observational study spanning a period of 3 months (August 2014 – April 2015) and involving 50 HIV-uninfected Sowetan women aged between 18 and 25 who provided questionnaire-directed answers investigating the frequency and nature of post-coital vaginal ‘cleansing’ practices. Do vaginal practices increase the risk of HIV acquisition, that is by causing low-level, but recurrent trauma to the genital tract? The aim of the study was to describe local practice. The authors note that South Africa’s overall HIV prevalence among 20–24 year-olds is 16% and in Gauteng where this study was performed, prevalence in the general population is 18%. Exposure to infection was high. On average, the study group recorded having sex 15.3 times per month with their main partner, having casual sex 10 times per month and having sex with a ‘new’ casual partner 3.6 times per month. Condom use was rare. However, this increased over the course of the study viz. 2% at baseline to 20% (main partner) and to 56% (casual partner) by the end of the study. No HIV infections occurred. Cleansing practices included washing the vagina with water (44%) and using fingers to facilitate cleaning (48%) and were more likely practised after inconsistent condom use or sex with a casual partner, *p* = 0.001. These practices decreased over the course of the study. Despite being asymptomatic, 40%, *n* = 20 women had positive baseline lab tests for a genital tract infection.

10. Kateule E, Kumar R, Mwakazanga D, Mulenga M, Daka V, Chongwe G. A cross-sectional study of the factors associated with male circumcision status among college youth in Ndola, Zambia. South Afr J HIV Med. 2019;20(1):a952. https://doi.org/10.4102/sajhivmed.v20i1.952

**Editor’s comment:** This report discusses the knowledge, attitudes and perceptions of 136 male Zambian students with regard to male circumcision and voluntary medical male circumcision (VMMC) in particular. A total of 63% of the students had been circumcised and most (96%) had taken the formal medical route viz. VMMC. This study has several limitations: cohort-bias, the observational nature of the data, self-reporting by the students and ‘predictable’ results, for example the circumcised students viewed the procedure as safe (aOR = 5.13, *CI* = 2.09–14.82), and effective in reducing viral transmission from infected women to uninfected men (aOR = 3.65, *CI* = 3.12–11.67). (Note the wide confidence intervals). The 2012–2015 national coverage of VMMC in Zambia was only 54% while the adult prevalence of HIV was 12.3% (ZAMPHIR fact sheet, December 2016). What is it that makes adult men complacent in the face of this epidemic? This study does not provide the answer but certainly begs the question.

11. Chakalisa U, Wirth K, Bennett K, et al. Self-reported risky sexual practices among adolescents and young adults in Botswana. South Afr J HIV Med. 2019;20(1):a899. https://doi.org/10.4102/sajhivmed.v20i1.899

**Editor’s comment: Recommended reading**. This is an important substudy of a cross-sectional, cluster-randomised Combination Prevention Project based in Botswana: the ‘YaTsie Project’. The aim of the parent study is to evaluate the impact of interventions on the prevention of HIV in that country. The aim of the substudy was to identify and characterise the risk-taking sexual activities that promote viral transmission. The findings of the substudy are not surprising: self-reported risk-taking sexual behaviour of adolescents and young adults *differs* between males and females. Subjects were aged 16–24 years. Of the 3380 study participants, *n* = 2311 reported being sexually active viz. women (65%) and men (35%). Enrolment took place from October 2013 to November 2015. Univariate and multivariate data underline the importance of the following markers of risk among women: inconsistent condom use, intergenerational sex (with male partners > 10 years older) and transactional sex among the poor. On the other hand, women were less likely than men to report being sexually active before 15 years, to use alcohol at or during intercourse and to report ≥ 2 (multiple) sexual partners in the preceding 12 months. Men living close to urban areas and those with internet access were at greater risk of being HIV-positive. **This paper is a must-read for health workers and administrators across southern Africa**. Success of HIV prevention has been elusive in this age group. Treatment as prevention will take us far. But papers such as this provide tools that communities can use to facilitate change.

12. Mukumbang FC, Van Wyk B, Van Belle S, Marchal B. ‘At this [adherence] club, we are a family now’: A realist theory-testing case study of the antiretroviral treatment adherence club, South Africa. South Afr J HIV Med. 2019;20(1):a922. https://doi.org/10.4102/sajhivmed.v20i1.922

**Editor’s comment: ‘**How successful are adherence clubs actually?’ This paper examines two adherence clubs associated with a provincial public health facility in the Western Cape (facility Y) and provides a theoretic explanation (‘realist evaluation’) as to how and why clubs work. The authors remind us that ‘only 62.3% of all people living with HIV (PLHIV) in South Africa are virally suppressed’, (www.hsrc.ac.za/uploads/pageContent/9234/FINALPresentationsfor17Julylaunch.pdf.) and that only 63.3% of infected South Africans are retained in the national South African HIV healthcare programme (Fox et al. PLoS Med 2018;15:30–43) Without a cure in sight, South Africa needs a long-term programme that delivers stronger numbers. Although much of the paper is taken up with providing a coherent thesis, the discussion and case evaluation provide practical steps to assist with improving outcomes from adherence clubs. Figure 4 in the article is a useful summary of the thesis.

13. Bisschoff C, Coulon J, Isaacs Z, et al. HIV testing at birth. Are we getting it right? South Afr J HIV Med. 2019;20(1):a951. https://doi.org/10.4102/sajhivmed.v20i1.951

**Editor’s comment**: This is a brief retrospective, descriptive, file-audit of births to HIV-positive mothers at the Mangaung University Community Health Centre, Bloemfontein, South Africa, during 2016. **A third of all the mothers treated at the clinic in 2016 tested HIV-positive**. A total of 428 babies were born to these mothers. Out of the infected mothers 7.3% were teenagers. Testing at birth (PCR) was conducted in 87.6% of the HIV-exposed infants of whom four (1.1%) were positive. While birth PCR testing levels are commendable, only *n* = 157 (36.7%) of exposed infants had the recommended 10-week follow-up HIV-PCR test. Almost all exposed infants (*n* = 427, 99.8%) were given nevirapine prophylaxis. Did any of the infected children start on ART? ‘No records were kept’. While prevention of mother-to-child transmission (PMTCT) has been a great success, gaps in care still exist. A third of the mothers in 2016 were HIV positive…!? Ouch!!

**July 2019**

14. Solomons DJ, Van der Merwe A, Esterhuizen TM, Crowley T. Factors influencing the confidence and knowledge of nurses prescribing antiretroviral treatment in a rural and urban district in the Western Cape province. South Afr J HIV Med. 2019;20(1):a923. https://doi.org/10.4102/sajhivmed.v20i1.923

**Editor’s comment: NIMART** stands for nurse-initiated and (nurse) managed antiretroviral treatment. This is a cross-sectional survey conducted among 77 NIMART nurses recruited from 29 healthcare centres in the Western Cape province of SA. The study covered both urban and rural nurses and aimed to identify factors influencing the nurses’ knowledge base and managerial or clinical confidence. Important limitations are noted by the authors: the cross-sectional and retrospective design, the small cohort, the large numbers of nurses who despite being NIMART ‘authorised’, nonetheless *refused* to participate in the study viz. *n* = 18 (25%) rural nurses and *n* = 22 (33%) urban nurses. Potential biases, for example the ‘self-completing’ of the questionnaires, may have led to further limitations. Nonetheless, many nurses (50%) indicated high levels of confidence with regard to the nursing aspects of HIV patient management and examination. But importantly, only 14% felt themselves to be expert enough in the day-to-day interaction with patients, and in particular, with the switching and stopping of ART. Contact with a ‘clinical mentor or clinician’ was limited for almost half (*n* = 36/77, 47%): once a week (*n* = 19), once a month (*n* = 14) and annually (*n* = 3). The replies of some are worrying: ‘no’ (*n* = 34, 44%), when asked ‘do you feel your workload is acceptable?’, and ‘no’ (*n* = 37, 48%), when asked ‘are you satisfied with your work conditions’. Not surprisingly, the study found that training, personal feedback, mentoring and seeing or caring for lots of patients had positive results with respect to knowledge and confidence. The small print is what worries me. NIMART-trained nurses are a precious asset to South Africa’s HIV response. I am worried because of those NIMART nurses who refused to participate and those who did, yet expressed unhappiness with their situation. How widespread are these attitudes and views?

15. Chateau AV, Dlova NC, Dawood H, Aldous C. Outcomes of Stevens–Johnson syndrome and toxic epidermal necrolysis in HIV-infected patients when using systemic steroids and/or intravenous immunoglobulins in Pietermaritzburg, South Africa. South Afr J HIV Med. 2019;20(1):a944. https://doi.org/10.4102/sajhivmed.v20i1.944

**Editor’s comment:** This retrospective study involving 36 HIV-positive patients reports the outcome of Stevens–Johnson syndrome (SJS), toxic epidermal necrolysis (TEN) and the SJS–TEN ‘overlap’ syndrome during the 18-month period, January 2010–July 2011. Short-term (3-day) oral steroids and intravenous immunoglobulins (IVIG) were used in all. Active debridement of bullae, de-roofing of blisters among others, was avoided in favour of careful skin cleansing. **Out of the 36 patients 32 were female. Sixteen were pregnant. Almost all (93.8**%**) were on nevirapine at the time of admission and the mean CD4 count of the group was 267 cells/mm³ (SD 60.6)**. Ten (27.8%) were also taking anti-tuberculosis drugs, isoniazid (*n* = 2) and rifafour (*n* = 8). One pregnant patient died. No adverse steroid-related events were identified. Unfortunately, the study has not provided more recent data. I would love to know if the disappearance of nevirapine from most ART programmes has resulted in the disappearance of these life-threatening skin conditions? Nevirapine is no longer a regular part of local and international ART guidelines. (Meintjes G, Moorhouse MA, Carmona S, et al. Adult antiretroviral therapy guidelines 2017. S Afr J HIV Med. 2017;18(1):a776. https://doi.org/10.4102/sajhivmed.v18i1.776)

16. Munderi P, Were E, Avihingsanon A, et al. Switching at low HIV-RNA-1 RNA into fixed-dose combinations: TDF/FTC/ RPV is non-inferior to TDF/FTC/EFV in first-line suppressed patients living with HIV. South Afr J HIV Med. 2019;20(1):a949. https://doi.org/10.4102/sajhivmed.v20i1.949

**Editor’s comment: Highly recommended**. This paper details the results of the SALIF study (SALIF = switching at low HIV-1 RNA into fixed-dose combinations). The study was conducted between August 2012 and October 2015 in five sub-Saharan countries viz. Cameroon, Kenya, Senegal, South Africa and Uganda and one Asian country, Thailand. It is a phase 3b, randomised, open-label, non-inferiority first-line ART switch-study that introduced RPV to virologically suppressed (HIV-RNA < 50 cp/mL) patients who had completed ± 12 months of either efavirenz (55%) or nevirapine (45%). The backbone Nucleoside reverse transcriptase inhibitors (NRTI) component of the regimen was tenofovir (TDF) + emtricitabine (FTC) before and after the switch. The RPV switch required the following: virological suppression (viral load < 50 cp/mL), CD4 count > 200 c/mm³, a normal baseline electrocardiograph (ECG) and the absence of concurrent tuberculosis (TB) therapy. Of the total cohort of 426 subjects, half (*n* = 211), that is the comparator arm, either continued with TDF+FTC+EFV throughout the study or switched to EFV from nevirapine (NVP) after an initial ± 12 months on TDF + FTC + NVP. The RPV arm, *n* = 213, switched to RPV + TDF + FTC having completed an initial 12 months on TDF + FTC + EFV. Both drug combinations were administered as single-tablet combination regimens (STRs). The RPV arm met the 48 week efficacy viz. ≥ 10% non-inferiority criteria and rate of virological failure requirements viz. viral suppression (< 400 cp/mL); RPV arm, *n* = 200/213 (93.8%) and EFV arm, *n* = 203/211 (96.2%). More subjects discontinued the study in the RPV arm (8%) as compared to the EFV arm (4.7%), (*n* = 27). This appeared to have been driven by an increase in adverse events (3.3% vs. 0.5%) in the RPV arm and an unanticipated closure of one of the study sites. The number of discontinuations is small. The increase in adverse events has not been previously reported in similar RPV versus EFV studies. Dr Moorhouse and Dr Cohen provide an Opinion Piece on RPV Use in South Africa in the SAJHIVMED of the 29th May this year. (See item no. 5 discussed earlier).

Moorhouse et al. focus on the limitations of RPV in first-line ART in SA viz. baseline viral loads are unchecked in the public sector, many needing to start ART in SA present with low CD4 counts < 200 c/mm³, many in SA are already undergoing TB (rifampicin) therapy and the recording of baseline QT-intervals in South Africans initiating ART is not routine. Nevertheless, Munderi’s paper suggests that a novel role for RVP, for example first-line switch studies, remains an option in those who satisfy the criteria. This is a thoughtful and well-written paper.

17. Lilian RR, Rees K, Mabitsi M, McIntrye JA, Struthers HE, Peters RPH. Baseline CD4 and mortality trends in the South African human immunodeficiency virus programme: Analysis of routine data. South Afr J HIV Med. 2019;20(1):a963. https://doi.org/10.4102/sajhivmed.v20i1.963

**Editor’s comment: Highly recommended.** This paper reviews HIV changes viz. in mortality and CD4 numbers at presentation, in South Africa from 2004 to 2016. The tables and figures provide a very clear window as to what is happening in this region. The University of Cape Town’s TIER.Net database provided the *n* = 203,131 and *n* = 101,814 anonymised patient records of the respective Johannesburg (JHB) and Mopani (MPI, Limpopo, rural) regions analysed. The paper focuses on mortality in relation to CD4 counts < 200 c/mm³. It also draws attention to the post-2013 decline in ART initiations in both regions – despite the fact that neither has yet achieved the 90-90-90 goals of the UNAID and the World Health Organization (WHO). In both regions, it is women who outnumber men with regard to ART initiations viz. 63–67% JHB and 68% MPI. In their analysis of the meaning of a low baseline, that is, CD4 count < 200 c/mm³ at ART initiation, this is the group with the highest early mortality after starting ART and *over a 5-year period*. The data are significant (*p* < 0.001) whether urban or rural. The risk is still present in the 2016/2017 data. The percentage of those initiating ART at these low levels remains high at this time viz. ± 40% in JHB and 35% in MPI. Who are the ones who are at greatest risk of initiating ART at low CD4 levels? Men, the elderly, the hospitalised. The authors make the point – not new – that these citizens of SA are not invisible to society. This is a very thought-provoking study. For those among us who teach medicine, this paper has robust data, excellent tables and figures and a great deal to talk about. **This paper is a must-read for all our HIV Clinicians’ Society members.**

18. Rossouw TM, Van Dyk G, Van Zyl G. Rapid emergence of resistance to antiretroviral treatment after undisclosed prior experience: A case report. South Afr J HIV Med. 2019;20(1):a965. https://doi.org/10.4102/sajhivmed.v20i1.965

**Editor’s comment:** This is a short case report of a 43-year-old female whose prior exposure to first-line ART (2012–2013) was revealed following failure of what had been believed to be the patient’s first exposure to ART in July 2014. Genotype testing at the commencement of ART in July 2014 failed to reveal viral mutations. However, these emerged after the (re)start of antiviral therapy. This report is a reminder that failure to suppress HIV after first-line therapy must trigger the possibility of prior exposure to ARVs in addition to inadequate adherence. A comprehensive medical history must *always* include questions about prior ART exposure.

